# Effect of short‐ versus long‐term serum glucose levels on early ischemic water homeostasis and functional outcome in patients with large vessel occlusion stroke

**DOI:** 10.1111/ene.16166

**Published:** 2023-11-28

**Authors:** Susan Klapproth, Lukas Meyer, Helge Kniep, Matthias Bechstein, Anna Kyselyova, Uta Hanning, Gerhard Schön, Leander Rimmele, Jens Fiehler, Gabriel Broocks

**Affiliations:** ^1^ Department of Diagnostic and Interventional Neuroradiology University Medical Center Hamburg‐Eppendorf Hamburg Germany; ^2^ Institute of Medical Biometry and Epidemiology University Medical Center Hamburg‐Eppendorf Hamburg Germany; ^3^ Department of Neurology University Medical Center Hamburg‐Eppendorf Hamburg Germany

**Keywords:** BGL, cerebral edema, functional outcome, NWU, stroke

## Abstract

**Background and purpose:**

In ischemic stroke, the impact of short‐ versus long‐term blood glucose level (BGL) on early lesion pathophysiology and functional outcome has not been assessed. The purpose of this study was to directly compare the effect of long‐term blood glucose (glycated hemoglobin [HbA1c]) versus serum BGL on early edema formation and functional outcome.

**Methods:**

Anterior circulation ischemic stroke patients who underwent mechanical thrombectomy after multimodal computed tomography (CT) on admission were analyzed. Endpoints were early ischemic cerebral edema, measured by quantitative net water uptake (NWU) on initial CT and functional independence at Day 90.

**Results:**

A total of 345 patients were included. Patients with functional independence had significantly lower baseline NWU (3.1% vs. 8.3%; *p* < 0.001) and lower BGL (113 vs. 123 mg/dL; *p* < 0.001) than those without functional independence, while HbA1c levels did not differ significantly (5.7% vs. 5.8%; *p* = 0.15). A significant association was found for NWU and BGL (*ß* = 0.02, 95% confidence interval [CI] 0.006–0.03; *p* = 0.002), but not for HbA1c and NWU (*ß* = −0.16, 95% CI −0.53–0.21; *p* = 0.39). Mediation analysis showed that 67% of the effect of BGL on functional outcome was mediated by early edema formation.

**Conclusion:**

Aggravated early edema and worse functional outcome was associated with elevated short‐term serum BGL, but not with HbA1c levels. Hence, the link between short‐term BGL and early edema development might be used as a target for adjuvant therapy in patients with ischemic stroke.

## INTRODUCTION

Acute ischemic stroke (AIS) is one of the leading causes of death worldwide [1]. Mechanical thrombectomy (MT) has been shown to improve functional outcomes in patients with AIS caused by a large vessel occlusion [2, 3]. The indication for MT has recently been extended to patients presenting in the unknown or extended time window from symptom onset [4, 5] and to patients with large ischemic stroke (i.e., those with low Alberta Stroke Program Early Computed Tomography Score (ASPECTS)/large ischemic core volume) [6, 7, 8, 9]. Currently, a major focus in stroke research and care is the search for adjunctive and complementary treatment options to further improve outcomes for stroke patients. The ESCAPE‐NA1 trial showed that nerinetide was of benefit to patients not receiving intravenous alteplase [10]. Currently, the CHARM trial is assessing the effects of administration of intravenous glibenclamide, an antidiabetic drug known to be associated with reduced ischemic edema formation in stroke, on functional outcome in AIS patients.

It is well known that elevated blood glucose is associated with poor functional outcome, increased mortality, and increased risk of symptomatic hemorrhage [11, 12, 13]. Many studies have discussed elevated blood glucose levels (BGL) as a potentially modifiable predictor of outcome, but data were mostly obtained from cohorts including patients not achieving recanalization. Similarly, it has been observed that glycated hemoglobin (HbA1c) levels are predictive of functional outcome following AIS [14]. However, only few studies have analyzed HbA1c and glucose levels in the context of patients receiving endovascular treatment or, in particular, the correlation of glucose levels with early ischemic changes and functional outcome. Furthermore, there are no studies addressing the role of long‐term blood glucose levels and their effect on aggravation of cerebral edema, although an association of serum glucose levels and edema formation has already been described [15]. Nevertheless, the association of short‐term versus long‐term BGL with outcome has important clinical relevance because it may be directly linked to the effect and benefit of any acute therapeutic BGL modification, or the utilization of further adjuvant treatment options. Early treatment and prevention of edema formation are crucial because this can lead to serious complications, resulting in a mortality rate of up to 80% [16]. The aim of this study was to investigate the associations of HbA1c and BGL with early brain edema using quantitative net water uptake (NWU) as an imaging biomarker. We hypothesized that HbA1c is a more accurate predictor of edema formation and functional outcome than serum BGL on admission.

## METHODS

### Patients

We retrospectively analyzed patients with AIS who had a large vessel occlusion (middle cerebral artery or distal internal carotid artery) treated at the local high‐volume university stroke center during the period January 2015 to January 2022. Anonymized data were recorded in accordance with ethical review board approval, with informed consent having been waived by our institutional review board (*Ethikkommission der Ärztekammer Hamburg* [WF0413]). The study was performed in accordance with the Declaration of Helsinki.

The a priori‐defined inclusion criteria of this study were: (i) AIS with large vessel occlusion of the distal internal carotid artery or M1 segment of the middle cerebral artery confirmed by multimodal computed tomography (CT) on admission with non‐enhanced CT, CT angiography and CT perfusion; (ii) visually evident early infarct lesion as indicated by ischemic hypoattenuation in admission non‐enhanced CT and/or lesion on CT perfusion with reduced cerebral blood flow; (iii) endovascular procedure performed according to the current guidelines, with documented modified Thrombolysis In Cerebral Infarctions (mTICI) score (an mTICI score ≥ 2b was defined as successful recanalization); (iv) absence of intracranial hemorrhage and preexisting infarctions in admission non‐enhanced CT; and (v) complete documentation of serum BGL on admission and HbA1c.

Baseline clinical characteristics and demographic information were extracted from the medical records. Functional outcome was extracted from the registry using modified Rankin Scale (mRS) scores after 90 days. History of diabetes mellitus was retrieved from clinical documentation. Elevated BGL was defined as ≥140 mg/dL according to the current guidelines of the Centers for Disease Control and Prevention [17, 18]. HbA1c thresholds were defined as <5.7% (normal), 5.7%–6.4% (elevated), and ≥6.5% (strongly elevated) [19, 20, 21].

The primary endpoint was early ischemic water homeostasis, defined using quantitative NWU as an imaging biomarker [15, 22, 23], which was assessed on baseline CT as described previously [24]. The secondary endpoint was functional independence, defined as mRS scores of 0–2 after 90 days.

### Imaging analysis

Data were anonymized and processed at the local imaging laboratory for blinded analysis. We used a standardized procedure to quantify early NWU in the ischemic core lesion at the time of admission imaging. This procedure is reported separately in detail elsewhere [22, 23, 24, 25]. Commercially available software (Analyze 11.0, Biomedical Imaging Resource, Mayo Clinic, Rochester, MN, USA) was used for analysis. In summary, quantitative NWU is an established densitometric method by which to assess ischemic edema formation on CT. The method used to determine quantitative NWU due to ischemic cerebral edema was originally described as an imaging biomarker for the assessment of lesion age and was subsequently extensively validated in further in vitro and in vivo studies [22, 23, 26]. In brief, the mean density of the core lesion is calculated on non‐enhanced CT after defining the region of interest (ROI) for analysis on CT perfusion parameter maps (defined as described by Minnerup et al.: Areas of reduced cerebral blood volume within the total hypoperfused lesion defined by time to drain maps) [23]. The density of the physiological tissue is calculated by mirroring the ROI with the contralateral hemisphere. Collateral status was assessed using an established five‐point scoring system by Souza et al. [27]. Scoring was performed by an experienced neuroradiologist and validated by a second experienced neuroradiologist [27]. Poor collaterals were defined as grade 0–2 and good collaterals as 3–4 according to Kim et al. [19]. ASPECTS were derived from clinical documentation. This rating is carried out regularly by an experienced neuroradiologist and reviewed by a second attending neuroradiologist. These scores were finally verified for accuracy in a consensus reading. Image analysis was performed blinded to clinical data.

### Statistical analysis

Continuous variables are presented as means or confidence intervals (CIs) of means, standard deviation (SD) or median and interquartile range (IQR). Kolmogorov–Smirnov tests were used to determine if the datasets were well modelled by a normal distribution. Patients with functional independence (mRS scores 0–2) were compared to patients with worse outcome (mRS scores 3–6) in Table 1 using Student's *t*‐tests (normal distribution) or Mann–Whitney *U*‐tests (non‐normal distribution). The association between baseline variables including BGL and HbA1c and early edema progression (i.e., quantitative NWU), was analyzed using multivariable linear regression, including collateral status, ASPECTS, age, and National Institute of Health Stroke Scale (NIHSS) score as covariables. All independent variables were tested for collinearity in advance. The α value of the slope was set at 0.05 level, and all reported results are two‐sided. The associations of baseline and treatment variables with clinical outcome were analyzed using univariable and multivariable logistic regression analysis, with backward selection, including collateral score, age, NIHSS score, ASPECTS, NWU, BGL, HbA1c level determined in the course, and recanalization status (Table 2). The dependent variable was functional independence (mRS score 0–2). Mediation analyses [28, 29] were used to evaluate to what extent the modification of edema formation by BGL changes explain the effect of BGL on outcome in patients undergoing MT. Mediation analysis was performed using a template described by Baron and Kenny [28], with BGL as a mediator variable. Sobel's and Monte Carlo tests were used to test for partial mediation, with Monte Carlo repetitions set at 500. The ratio of indirect effect size to total effect was calculated. Three pathways were tested in advance in order to perform the mediation analysis: (i) the association of BGL with mRS score at Day 90; (ii) the association of BGL with NWU; and (iii) the association of NWU with mRS score at Day 90, controlling for treatment type. After confirming these associations, a mediation (indirect effect) may be established through estimation of the direct causal relationship. Pathways were tested using univariable and multivariable linear regression analysis. BGL was log‐transformed (log+1) to satisfy a linear model (residual distribution was normal and homoscedasticity of the data was preserved). A statistically significant difference was accepted at a *p* value of less than 0.05. Analyses were performed using Stata/MP 17.0 (Stata Corp, College Station, TX, USA).

**TABLE 1:** Patient characteristics.

Baseline characteristics	Functional independence	mRS score 3–6	*p* value
Subjects, *n* (%)	121 (35)	224 (65)	
Baseline variables			
Age, median (IQR) years	70 (57–79)	78 (68–84)	<0.001
Female sex, *n* (%)	(55)	(44)	0.05
Admission NIHSS score, median (IQR)	9 (7–10)	17 (14–20)	<0.001
Time onset to imaging, median (IQR) h	2.8 (1.3–5.6)	3.1 (1.7–6.1)	0.09
ASPECTS, median (IQR)	8 (7–9)	7 (5–8)	<0.001
NWU, median (IQR)	3.1 (1.3–4.5)	8.3 (6.6–10.1)	<0.001
Blood glucose, median (IQR) mg/dL	113 (101–131)	123 (107–155)	<0.001
High blood glucose (>140 mg/dL), *n* (%)	34 (28)	103 (46)	<0.001
HbA1c, median (IQR) %	5.7 (5.6–5.9)	5.8 (5.5–6.5)	0.25
High HbA1c (>6.4%), *n* (%)	64 (53)	151 (67)	<0.001
Treatment and endpoints			
IVT administration, *n* (%)	83 (69)	114 (51)	0.002
mTICI score 2b/3, *n* (%)	108 (89)	154 (69)	<0.001
mRS score, median (IQR)	1 (0–2)	5 (4–6)	<0.001

Abbreviations: ASPECTS, Alberta Stroke Program Early CT Score; HbA1c, glycated hemoglobin; IQR, interquartile range; IVT, intravenous thrombolysis; mTICI, modified Thrombolysis in Cerebral Infarction; mRS, modified Rankin Scale; NIHSS, National Institute of Health Stroke Scale.

**TABLE 2:** Multivariable linear regression analysis investigating the association of early edema formation with baseline variables.

	*ß*	95% CI	*p* value
BGL model			
Age	0.04	0.02–0.06	<0.001
ASPECTS	−0.62	−0.78 to −0.46	<0.001
Time from onset	−0.005	−0.08 to 0.08	0.97
Collateral score	−1.85	−2.42 to −1.26	<0.001
Occlusion location	−0.59	−0.90 to −0.29	<0.001
BGL	0.01	0.005 to 0.02	0.001
HbA1c model			
Age	0.02	−0.007 to 0.05	0.14
ASPECTS	−0.47	−0.16 to 0.07	<0.001
Time from onset	−0.05	−0.16 to 0.07	0.43
Collateral Score	−2.36	−3.18 to −1.54	<0.001
Occlusion location	−0.51	−0.92 to −0.10	0.27
HbA1c	0.17	−0.14 to 0.48	0.27

Abbreviations: ASPECTS, Alberta Stroke Program Early CT Score; BGL, blood glucose level; CI, confidence interval; HbA1c, glycated hemoglobin; IQR, interquartile range; mTICI, modified Thrombolysis in Cerebral Infarction; mRS, modified Rankin Scale; NIHSS, National Institute of Health Stroke Scale.

## RESULTS

**FIGURE 1:**
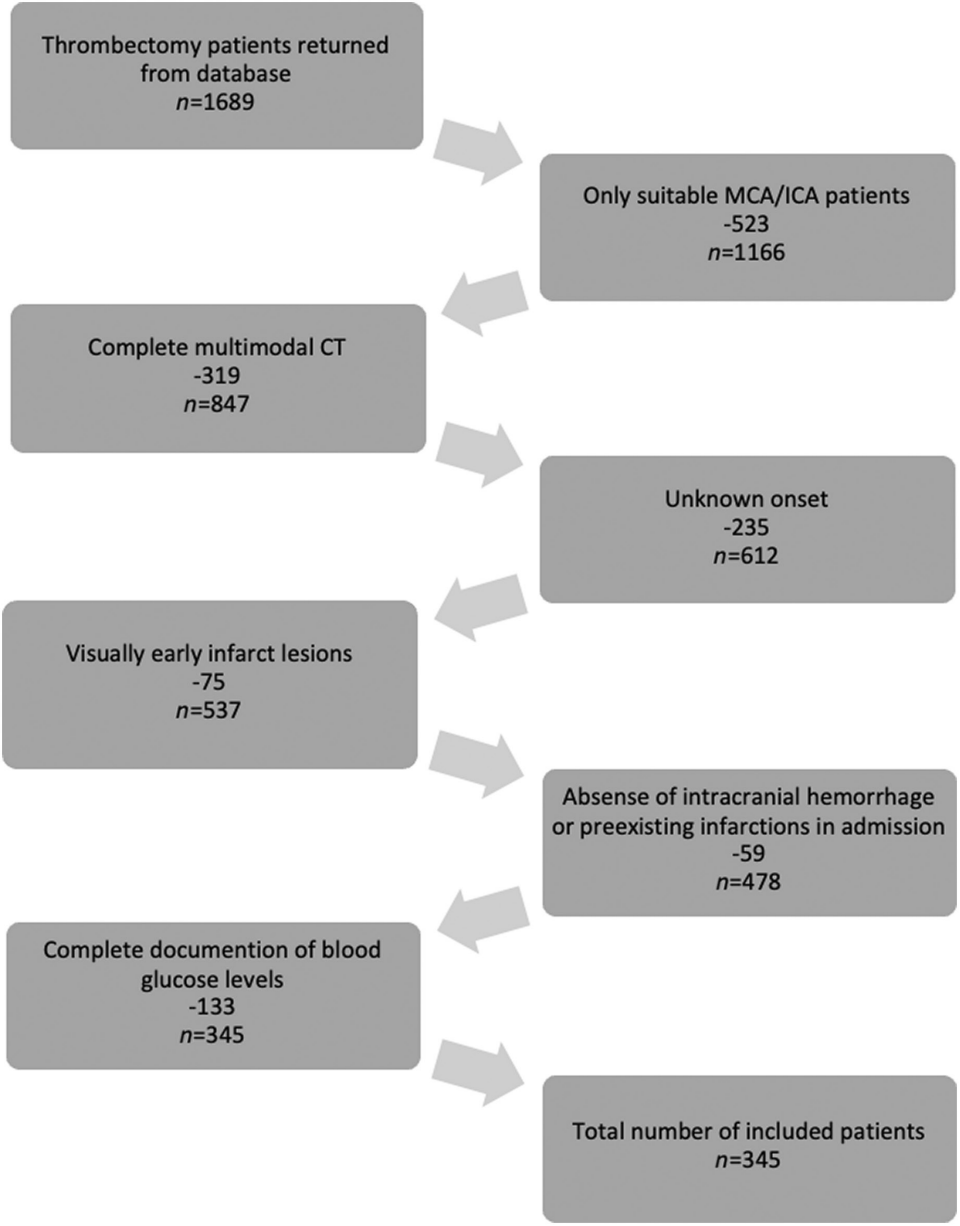
Flow chart of patient inclusion. ICA, internal carotid artery; MCA, middle cerebral artery.

A total of 345 patients were included, of whom 47% (*n* = 162) were women (Figure 1). The mean (SD) age was 74 (13) years, and the median (IQR) NIHSS score was 14 (9–18). The median (IQR) ASPECTS was 7 (6–9), and the median (IQR) time from onset to imaging was 3.4 (2.1–6.5) h. The median (IQR) collateral score was 3 (2–3). The median (IQR) BGL was 120 (104–143) mg/dL and the median (IQR) HbA1c was 5.7% (5.5–6.1%). A total of 152 patients (44%) had elevated BGL on admission and 30% had BGL > 200 mg/dL. In all, 190 patients (55%) received intravenous treatment with alteplase, and the rate of successful recanalization was 76%.

### Association of blood glucose levels and early edema formation

**FIGURE 2:**
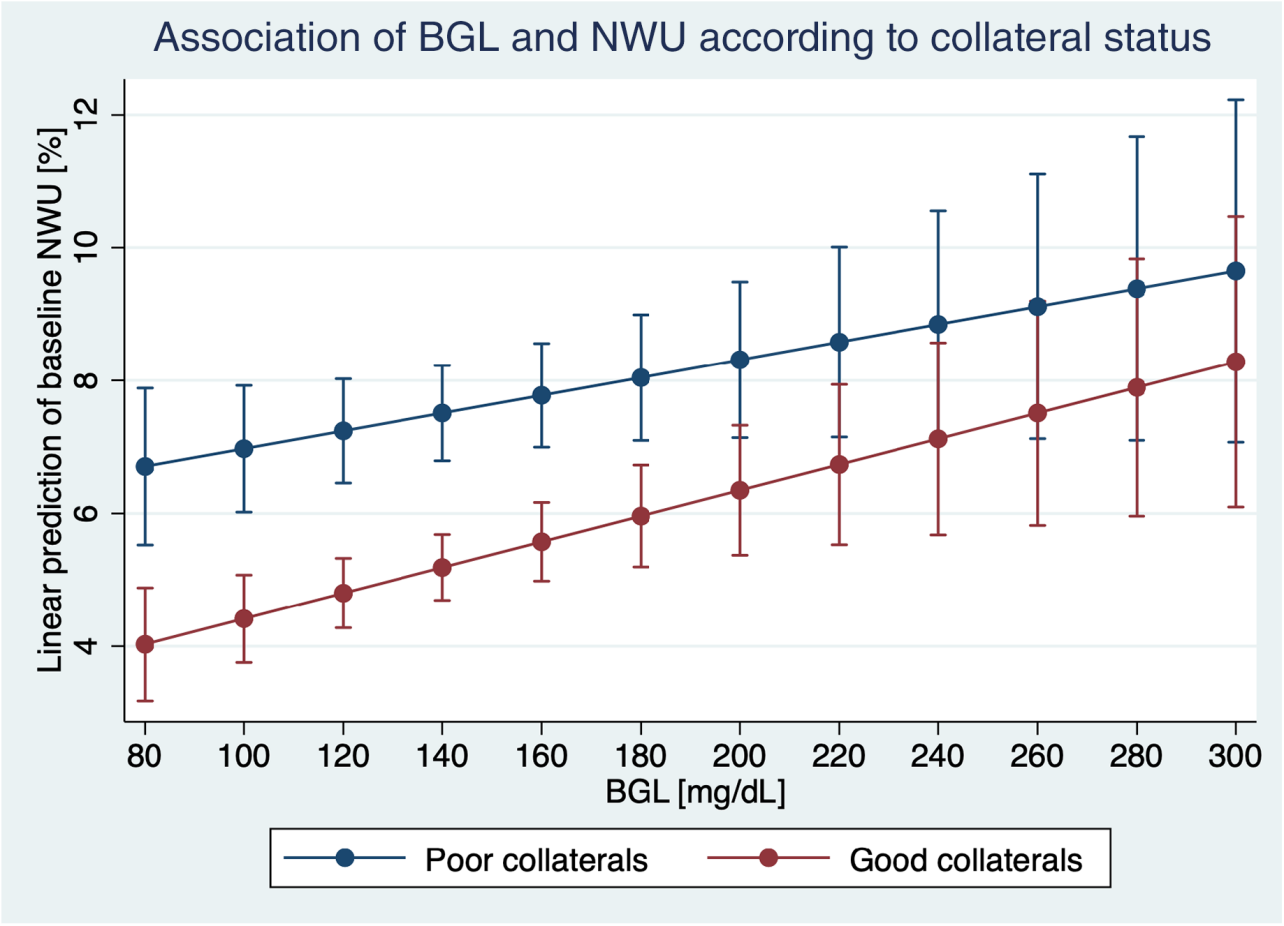
Multivariate linear regression analysis showing the impact of blood glucose level (BGL; *x*‐axis) on ischemic brain edema (net water uptake [NWU]; *y*‐axis) based on collateral score (good collateral status 3–4 vs. poor collateral status 0–2).

The median NWU for patients with elevated HbA1c (>6.4%) was higher compared to patients with lower HbA1c (6.8% vs. 5.4%; *p* = 0.0003). Similarly, the median NWU was significantly higher in patients with higher BGL (>140 mg/dL) compared to patients with lower BGL (7.6% vs. 5.5%; *p* < 0.0001). A significant correlation between BGL and NWU was observed (*r* = 0.19, *p* < 0.0001), but not for HbA1c and NWU (*r* = 0.06, *p* = 0.26). In multivariable linear regression analysis, age (*ß* = 0.04, *p* < 0.001), ASPECTS (*ß* = −0.62, *p* < 0.001), collateral status (*ß* = −1.85, *p* < 0.001), occlusion location (*ß* = −0.59, *p* < 0.001) and BGL (*ß* = 0.01, *p* = 0.001) were independently associated with NWU (Figure 2). There was no association between HbA1c level and early NWU (*p* = 0.27; Table 2).

### Prediction of functional outcome

Comparing patients with functional independence to patients with mRS scores 3–6 at Day 90, we observed significant differences between the two groups in age, NIHSS score and ASPECTS (Table 1). Furthermore, patients with functional independence had lower early NWU (3.1% vs. 8.3%; *p* < 0.001) and lower BGL on admission (113 mg/dL vs. 123 mg/dL; *p* < 0.001). HbA1c levels were not different between the two groups, but the proportion of patients with significantly elevated HbA1c (>6.4%) was higher in patients with poor outcome (67% vs. 53%; *p* < 0.001). Patients with functional independence had a significantly better collateral score (median score 3 in both groups; *p* < 0.0001). In multivariable logistic regression analysis, an independent association with functional outcome was observed for BGL (adjusted odds ratio [aOR] 0.98, 95% CI 0.98–0.99; *p* = 0.003), collateral status (aOR 2.84, 95% CI 1.59–5.05; *p* < 0.001) and successful recanalization (aOR 3.45, 95% CI 1.83–6.53; *p* < 0.001). Further independent predictors were age, NIHSS, ASPECTS, and occlusion location.

### Mediation analysis

For the mediation analysis, all requirements according to Baron and Kenny [28] were fulfilled. Regression analysis of the direct path confirmed the significant associations of the independent predictors and the mediators with the probability of functional independence. Furthermore, significant coefficients were observed for the regression of the mediator on the independent predictors. Sobel's test and the Monte Carlo test were significant. All effect metrics were significant at *p* < 0.01. Mediation analysis using NWU as the mediator indicating edema formation showed that 67% of the effect of BGL on mRS score at Day 90 was mediated by NWU (indirect effect 0.6, 95% CI 0.3–0.8, *p* < 0.001; total effect 0.9, 95% CI 0.5–1.2, *p* < 0.001).

## DISCUSSION

The purpose of this study was to investigate the effect of HbA1c levels on the formation of early ischemic edema using a quantitative imaging biomarker directly compared to the effect of serum BGL. The main findings of this study were that (i) both serum BGL and HbA1c level were associated with functional outcome, although only BGL showed an independent association with mRS scores at Day 90; (ii) serum BGL was independently associated with the degree of early edema formation, while HbA1c was not associated with quantitative NWU, (iii) the association of elevated early edema formation and higher BGL was pronounced in patients with a lower HbA1c, highlighting the effect of short‐term hyperglycemia, (iv) early edema formation was higher in patients with worse collaterals; however, the effect of increasing BGL on edema formation was more distinct in patients with good collaterals, and (v) mediation analysis showed that a significant proportion of the effect of BGL on functional outcome (i.e., 67%) was mediated by increasing early edema formation.

**FIGURE 3:**
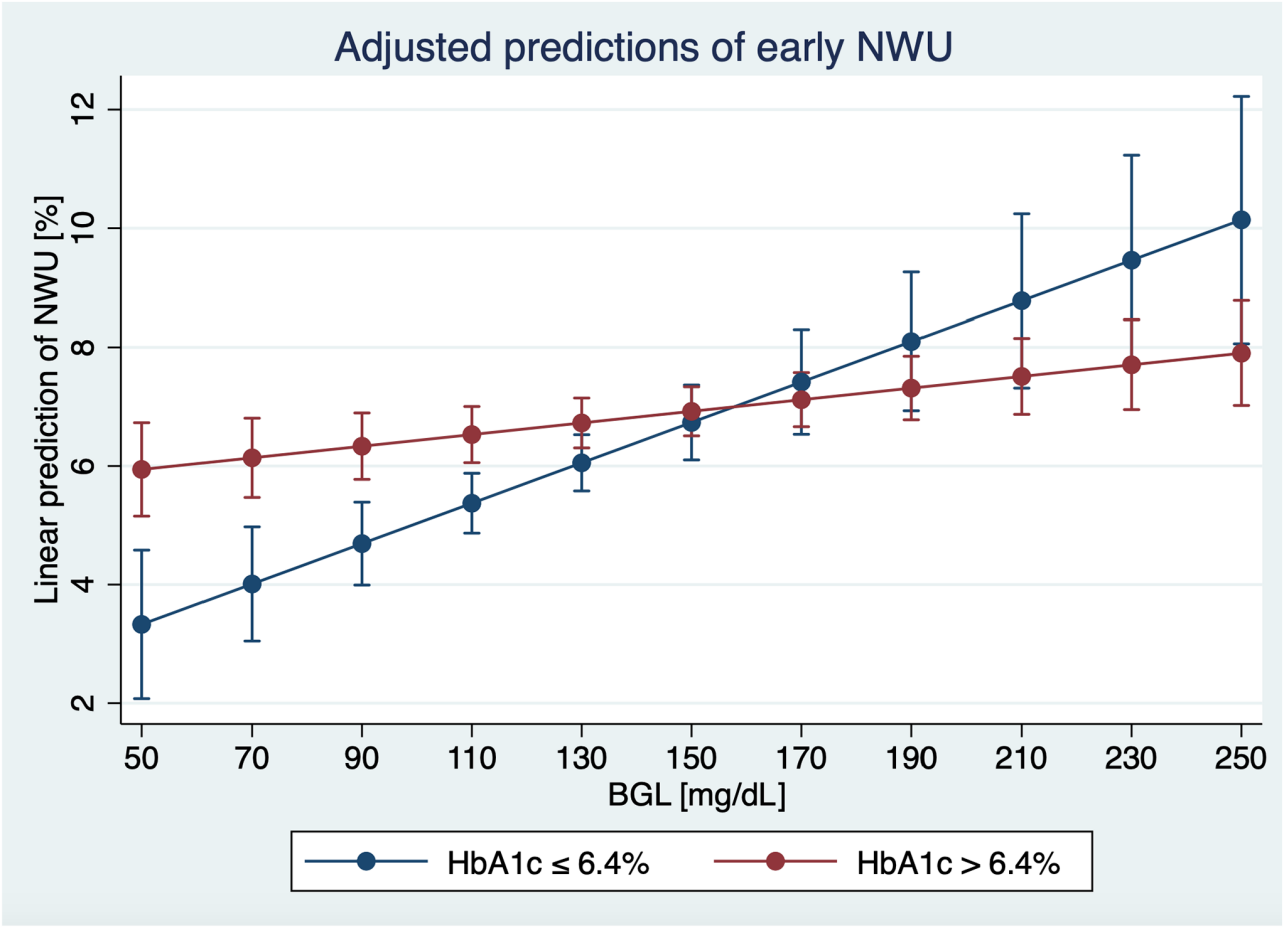
Multivariate linear regression analysis showing the impact of blood glucose levels (BGL; *x*‐axis) on ischemic brain edema (net water uptake [NWU]; *y*‐axis) based on long‐term BGL. HbA1c, glycated hemoglobin.

In ischemic stroke patients, the assessment of blood sugar profiles including serum BGL and HbA1c levels is a standard of care. However, there are no specific acute implications of these parameters during patient admission or treatment. It is known that approximately 23%–53% of stroke patients have prediabetes, while the proportion of patients with diabetes is 14%–46% [30]. The increasing trends in prediabetes and diabetes prevalence will likely result in higher stroke burden in the future [30]. In this study, we observed a high variation in serum BGL on admission, with a median BGL of 120 mg/dL, but a 5%–95% percentile of 90–226 mL/dL, and nearly half of the patients had BGL of >140 mg/dL, and approximately a third of the patients had BGL >200 mg/dL. Correspondingly, quantitative NWU on admission ranged between 0% and 13.4% (5%–95% percentile) showing a significant correlation to BGL, but not to HbA1c levels. As illustrated in Figure 3, the effect of higher BGL on the formation of early ischemic edema was pronounced in patients with lower HbA1c levels, emphasizing the impact of short‐term BGL elevation. Patients with higher HbA1c levels might show a higher degree of physiological adaptation to a state of elevated BGL and, hence, lower dynamic ischemic lesion progression during acute ischemia [31]. This association might also be a factor contributing to the observation that younger patients may have comparably higher ischemic edema formation than older patients [32].

In the past, it has been observed that edema formation is associated with short‐term hyperglycemia in patients with or without known diabetes [33]. It has also been observed that the deleterious effect of BGL on outcome is directly linked to edema formation [15, 34]. Currently, a major focus in stroke research is the identification of further targets for adjuvant therapy. Hence, potentially modifiable parameters influencing functional outcomes or the response to reperfusion are being intensively investigated. Previous studies investigated the effect of insulin application on modifying BGL on admission. However, intensive insulin treatment did not improve functional outcomes compared to standard treatment. Of note, a high proportion of the included patients had mild to moderate stroke (mean total NIHSS score of 7) [35], which is lower than the median NIHSS score of typical large vessel occlusion patients (the median NIHSS score in the MR CLEAN cohort was 17 [36]). More importantly, endovascular recanalization was performed in only 13% of patients [35]. Therefore, the combination of adjuvant treatment including BGL modification with reperfusion might lead to better outcomes. There is currently no standardized approach for monitoring the treatment effect on ischemic brain edema following the administration of adjuvant neuroprotection. Quantitative NWU is an established imaging biomarker based on previous research encompassing theoretical studies, in vitro experiments, and clinical in vivo investigations, which collectively established a direct connection between the decrease in radiodensity (hypoattenuation) of an infarct lesion and its proportional increase in volume caused by edema [23, 25].

Further studies have compared whether the administration of uric acid may improve functional outcome in patients with acute stroke and hyperglycemia. Uric acid was shown to be more effective than placebo in reducing infarct growth in patients in the upper serum glucose tertile [37]. Moreover, sulfonylureas, especially glibenclamide, may reduce lesional water uptake as well as mass effect after major hemispheric infarction [38]. The CHARM trial is currently evaluating the effect of intravenous glibenclamide in patients with large infarctions (i.e., ASPECTS 1–5, lesion volume 80–300 mL [39]). However, it is important to note that, for patients undergoing endovascular treatment, inclusion in this trial is based on post‐thrombectomy diffusion‐weighted magnetic resonance imaging. At this timepoint, edema formation may have already progressed significantly, particularly in patients with hyperglycemia. Hence, BGL modification immediately on admission, accompanied by timely administration of neuroprotective agents directly after baseline admission imaging, may lead to lower edema formation, and hence, better response to recanalization and functional outcome. Finally, the association of BGL with early neurological improvement should be investigated, particularly with regards to the “stunned brain phenomenon” [40].

Limitations of this study include the relatively low number of patients due to strict inclusion and exclusion criteria. Furthermore, this was a retrospective analysis which therefore requires further prospective validation. In addition, hyperglycemia after stroke is a dynamic process, therefore single values may not be sufficient to capture the full complexity of the ischemic brain.

In conclusion, acute hyperglycemia was independently associated with aggravated early edema formation and worse functional outcome, while there were no significant associations between HbA1c levels and edema formation. The link between short‐term serum glucose and early edema formation might therefore be a target for adjuvant stroke therapy.

## AUTHOR CONTRIBUTIONS

Susan Klapproth, Gabriel Broocks, Lukas Meyer, Anna Kyselyova, Uta Hanning and Jens Fiehler have contributed to the conception and design of the study. Susan Klapproth, Gabriel Broocks, Matthias Bechstein, Helge Kniep, Anna Kyselyova, Uta Hanning, Jens Fiehler and Gerhard Schön contributed to the acquisition and analysis of data. Susan Klapproth, Gabriel Broocks, Matthias Bechstein, Gerhard Schön, Anna Kyselyova and Jens Fiehler contributed to drafting a significant portion of the manuscript.

## FUNDING INFORMATION

None.

## CONFLICT OF INTEREST STATEMENT

Prof. Fiehler declares research support from the German Ministry of Science and Education (BMBF and BMWi), the German Research Foundation (DFG), the European Union, *Hamburgische Investitions‐ und Förderbank* (IFB), Medtronic, Microvention, Philips and Stryker, and has served as a consultant for Acandis, Boehringer Ingelheim, Cerenovus, Covidien, Evasc Neurovascular, MD Clinicals, Medtronic, Medina, Microvention, Penumbra, Route92, Stryker and Transverse Medical.

## Data Availability

The data that support the findings of this study are available from the corresponding author upon reasonable request.
